# The Use of Exome Genotyping to Predict Pathological Gleason Score Upgrade after Radical Prostatectomy in Low-Risk Prostate Cancer Patients

**DOI:** 10.1371/journal.pone.0104146

**Published:** 2014-08-05

**Authors:** Jong Jin Oh, Seunghyun Park, Sang Eun Lee, Sung Kyu Hong, Sangchul Lee, Gheeyoung Choe, Sungroh Yoon, Seok-soo Byun

**Affiliations:** 1 Department of Urology, Seoul National University Bundang Hospital, Seongnam, Korea; 2 Department of Electrical and Computer Engineering, Seoul National University, Seoul, Korea; 3 School of Electrical Engineering, Korea University, Seoul, Korea; 4 Departments of Pathology, Seoul National University Bundang Hospital, Seongnam, Korea; The Chinese University of Hong Kong, Hong Kong

## Abstract

**Background:**

Active surveillance (AS) is a promising option for patients with low-risk prostate cancer (PCa), however current criteria could not select the patients correctly, many patients who fulfilled recent AS criteria experienced pathological Gleason score upgrade (PGU) after radical prostatectomy (RP). In this study, we aimed to develop an accurate model for predicting PGU among low-risk PCa patients by using exome genotyping.

**Methods:**

We genotyped 242,221 single nucleotide polymorphisms (SNP)s on a custom HumanExome BeadChip v1.0 (Illuminam Inc.) in blood DNA from 257 low risk PCa patients (PSA <10 ng/ml, biopsy Gleason score (GS) ≤6 and clinical stage ≤T2a) who underwent radical prostatectomy. Genetic data were analyzed using an unconditional logistic regression to calculate an odds ratio as an estimate of relative risk of PGU, which defined pathologic GS above 7. Among them, we selected persistent SNPs after multiple testing using FDR method, and we compared accuracies from the multivariate logistic model incorporating clinical factors between included and excluded selected SNP information.

**Results:**

After analysis of exome genotyping, 15 SNPs were significant to predict PGU in low risk PCa patients. Among them, one SNP – rs33999879 remained significant after multiple testing. When a multivariate model incorporating factors in Epstein definition – PSA density, biopsy GS, positive core number, tumor per core ratio and age was devised for the prediction of PGU, the predictive accuracy of the multivariate model was 78.4% (95%CI: 0.726–0.834). By addition the factor of rs33999879 in aforementioned multivariate model, the predictive accuracy was 82.9%, which was significantly increased (p = 0.0196).

**Conclusion:**

The rs33999879 SNP is a predictor for PGU. The addition of genetic information from the exome sequencing effectively enhanced the predictive accuracy of the multivariate model to establish suitable active surveillance criteria.

## Introduction

Active surveillance (AS) of prostate cancer (PCa) with delayed intervention represents an attractive management option, as it delays and possibly avoids the morbidity and potential mortality associated with radical prostatectomy (RP) or various radiotherapy alternatives [1]–[2]. Despite the promising results of several major surveillance cohorts, and its 10-year disease specific survival of 97–100% [3], the estimation of whether patients should be actively treated for low-risk PCa remains controversial, as multiple studies have reported that a considerable proportion of men qualifying for AS have aggressive tumor features at the time of RP [4]–[5]. Therefore, a well-established selection criterion among the PCa patients is important. Epstein et al. [6] developed a set of criteria for the prediction of clinically insignificant PCa (CIPC) before definitive treatment. As with Epstein's criteria, the National Comprehensive Cancer Network, (NCCN), defined very low-risk PCa as that with prostate-specific antigen (PSA) <10 ng/ml, PSA density ≤0.15 ng/ml/cm^3^, clinical stage ≤T1c, Gleason score (GS) ≤6, numbers of positive cores ≤2, and cancer involvement per core ≤50% [7]–[8]. These criteria of very low-risk PCa are currently widely used in the selection of patients for AS [9]. However even these criteria are not ideal, as 20% of patients who fulfilled these criteria had unfavorable pathological PCa characteristics (pathologic GS ≥7 or pathologic stage ≥T3) at RP [10]. Other studies have shown 24–48.6% pathological Gleason score upgrade (PGU) which was defined pathological GS 7 or higher, or upstaging after RP, among men who fulfilled the criteria for CIPC [10]–[11]. Therefore, many studies have emphasized the importance of novel molecular biomarkers to predict unfavorable pathological outcomes among men with clinically non-aggressive PCa. Such a biomarker might be act as an appropriate selection criteria for AS. Therefore, intensive genomic research is currently under way to identify molecular markers that can predict the outcome of PCa [12].

In the present study, we analyzed the genetic variants, which were significantly associated with PGU in low-risk PCa patients, with the use of exome sequencing, and we applied this genetic information to a clinical model to predict PGU, incorporating various factors, including the Epstein criteria. Our aim in this study was to identify a biomarker which has additional predictive accuracy to select appropriate patients for AS.

## Materials and Methods

### Ethics statement

The study was approved by our institutional review board, Seoul National University Bundang Hospital Institutional review board (IRB number: B-1312/232-302) and follows the rules atated in the Declaration of Helsinki. All participants gave written informed consent and were reimbursed for their participation.

### Study population

After obtaining institutional review board approval, 1002 PCa patients were enrolled in this study from November 2003 to July 2013. Blood specimens were collected prospectively from all patients. We excluded patients who underwent neoadjuvant hormone or radiation therapy, underwent prostate biopsy at another institution, and underwent prostate biopsy with <12 cores taken. To find factors that influence PGU low-risk PCa patients, (PSA <10 ng/ml, biopsy GS 6 and clinical stage ≤T2a), who underwent RP, were included in this analysis. Accordingly, 257 patients were enrolled, with complete records of serum PSA, clinical stage, biopsy GS, number of positive cores, cumulative length of the cores in all prostate biopsy cores, and pathological outcomes available. The 257 patients were stratified into two groups according to presence of PGU.

### Pathological Evaluation

Transrectal ultrasound (TRUS)-guided multi-core (≥12) biopsies were taken from all men using an automatic firing mechanism. The prostate was biopsied near the base, mid-gland, and apex, bilaterally, with at least six biopsies per side. Thus, 12 baseline biopsy cores were taken in all men, and additional biopsies were taken to include suspicious appearing lesions if needed. All RP specimens were processed according to the Stanford protocol [13]. All biopsy and RP specimens underwent pathological analysis by a single genitourinary pathologist (G.C.). PGU was defined by pathological GS of 7 or higher.

### Genotyping and quality control

Study samples were processed on the HumanExome BeadChip 12v1-1 (Illumina, Inc., San Diego, CA), which includes 242,901 markers focused on protein-altering variants. Details about single nucleotide polymorphism (SNP) content and selection strategies can be found at the exome array design webpage (http://genome.sph.umich.edu/wiki/Exome_Chip_Design).

Genotype calling was carried out using Illumina's GenTrain version 2.0 clustering algorithm with the GenomeStudio software (V2011.1). Cluster boundaries were determined using Illumina's standard cluster file. After additional visual inspection of SNPs with a call rate of <0.99, and SNPs with minor allele frequency of <0.002, 242,186 of 242,901 (99.71%) attempted markers were successfully genotyped, with a call rate of >95% (average call rate 99.98%). In total, 1,008 of 1,009 (99.9%) individuals were successfully genotyped (call rate >98%). For the 242,186 SNPs that passed quality control, genotype concordance among the 104 blind duplicate sample pairs was 99.998%. One individual per pair of six known twin pairs and six unexplained apparent duplicates were excluded. We carried out principal components analysis (PCA) twice, once excluding HapMap samples to identify population outliers, and then including HapMap samples to help interpret outliers. To avoid artifactual results due to family relatedness, we computed principal components using SNP loadings estimated from a subset of 7,304 not-close-relatives. We defined close relatives as ones for whom the estimated genome-wide identical-by-descent (IBD) proportion of alleles shared was >0.10. We estimated IBD sharing using PLINK's “-genome” option38, and carried out PCA using SMARTPCA37 on a linkage-disequilibrium-pruned set of 22,464 autosomal SNPs. These were obtained by removing large-scale high-LD regions, SNPs with a MAF <0.01, or SNPs with HWE P value <10–6, and carrying out LD pruning using the PLINK option: “–indep-pairwise 50 5 0.2”. Inspecting the first 10 PCs, we identified 12 population outliers, 9 of whom had self-reported non-Finnish ancestry; we excluded these 12 individuals from subsequent analysis.

### SNP analysis of exome sequencing

SNP genotype frequencies were examined for Hardy-Weinberg equilibrium (HWE) using the χ^2^ statistic, and all were found to be consistent, (P>0.05), with HWE among Korean controls. Data were analyzed using an unconditional logistic regression to calculate an odds ratio (OR) as an estimate of the relative risk of PGU associated with SNP genotypes. To determine the association between the genotype and haplotype distributions, a logistic analysis was performed controlling for age (continuous value) as covariate to eliminate or reduce any confounding factors that might influence the findings. Lewontin's D′ (|D′|) and the LD coefficient r^2^ were examined to measure linkage disequilibrium between all pairs of biallelic loci [14]. The haplotypes were inferred from the successfully genotyped SNPs using PHASE algorithm ver. 2.0 [15], using SAS version 9.1 (SAS Inc., Cary, NC, USA). The effective number of independent marker loci was calculated to correct for multiple testing, using the software SNPSpD (http://www.genepi.qimr.edu.au/general/daleN/SNPSpD/), which is based on the spectral decomposition (SpD) of matrices of pair-wise LDs between SNPs [16]. The resulting number of independent marker loci (23.1), was applied to correct for multiple testing. All p-values from the results were corrected for multiple testing by controlling for the false discovery rate (FDR) [17].

### Statistical analysis

A total of 257 low-risk PCa patients were stratified into two groups according to PGU. When comparing patients with and without PGU, we assessed the difference in clinicopathological profiles of patients using the chi-squared test, Fisher's exact test, and the Mann–Whitney test. Multivariate logistic regression with adjusting Epstein's clinical factors such as PSA density, clinical stage, number of positive biopsy core, percentage of tumor in a core, and age, was performed to identify an independent predictor of PGU. Predictive accuracy for the aforementioned multivariate logistic regression model was assessed with receiver operating characteristics–derived area under the curve (AUC) analysis. Another multivariate logistic regression model was built with the addition of genetic information derived from the exome sequencing, predictive accuracy was assessed by same method. The two AUCs were compared via a Mantel-Haenszel test. The SPSS software package version 15.0 (Statistical Package for Social Sciences, Chicago, IL, USA) and Medicalc software version 11 (Mariakerke, Belgium) was used for statistical analysis. A 2-tailed P<0.05 was considered significant for all analyses.

## Results

Among the 257 low-risk PCa patients, 203 patients (78.9%) showed PGU. The patient's characteristics according to PGU are described in Table 1. The 257 patients in the PGU group had higher PSA density, smaller prostate volume, higher positive core percentage, and higher tumor percentage in cores, than the 54 patients in the no PGU group.

**Table 1:** Clinicopathological characteristics of patients with and without pathologic Gleason score upgrading following radical prostatectomy.

	Total	Pathologic Gleason score		
Variables		6	≥7	p value
Number	257	54	203	
Age				0.086
Mean ± SD	64.77±7.18	63.06±8.50	65.23±6.74	
Median (range)	66 (43–79)	65 (43–75)	66 (45–79)	
Body mass index				0.783
Mean ± SD	24.18±2.60	24.27±2.78	24.16±2.69	
Median (range)	24.09 (14.12–33.61)	24.10 (20.76–31.89)	24.04 (14.12–33.61)	
PSA (ng/ml)				0.515
Mean ± SD	5.67±2.05	5.50±2.29	5.71±1.98	
Median (range)	5.58 (1.12–9.96)	4.96 (1.41–9.96)	5.61 (1.12–9.90)	
PSAD (ng/ml^2^)				<0.001
Mean ± SD	0.17±0.09	0.14±0.06	0.18±0.09	
Median (range)	0.16 (0.02–0.56)	0.12 (0.04–0.33)	0.17 (0.02–0.56)	
TRUS volume (ml)				<0.001
Mean ± SD	36.40±14.11	42.40±15.78	34.80±13.23	
Median (range)	33.60 (10.80–130.00)	42.60 (22.00–130.00)	32.00 (10.80–102.00	
Clinical stage (%)				0.207
T1	200 (77.8%)	43 (79.6%)	157 (77.3%)	
T2a	57 (22.2%)	11 (20.4%)	46 (22.7%)	
No. total cores sampled at biopsy (%)				0.101
12	164 (63.81%)	30 (55.6%)	134 (66.0%)	
≥13	93 (36.19%)	24 (44.4%)	69 (34.0%)	
Mean percent of positive cores				<0.001
Mean ± SD	19.60±14.21	12.51±9.78	21.58±14.64	
Median (range)	16.67 (5.56–83.33)	8.33 (5.56–58.33)	16.67 (6.67–83.33)	
Mean maximum tumor length in a core				<0.001
Mean ± SD	0.32±0.26	0.20±0.18	0.36±0.26	
Median (range)	0.20 (0.02–1.40)	0.10 (0.03–0.70)	0.30 (0.02–1.40)	
Mean maximum percent of tumor length in a core				<0.001
Mean ± SD	21.35±17.27	13.73±11.98	23.32±17.90	
Median (range)	16.67 (1.44–90.0)	11.98 (2.00–50.0)	17.90 (1.44–90.00)	
Extracapsular extension	23 (8.95%)	1 (1.85%)	22 (10.84%)	0.028
Seminal vesicle invasion	1 (0.39%)	0	1 (0.49%)	0.793
Positive surgical margin	39 (15.18%)	6 (11.1%)	33 (16.26%)	0.373

Abbreviations: PSA: prostate specific antigen; PSAD: prostate specific antigen density; TRUS: transrectal ultrasound.

**Figure 1:**
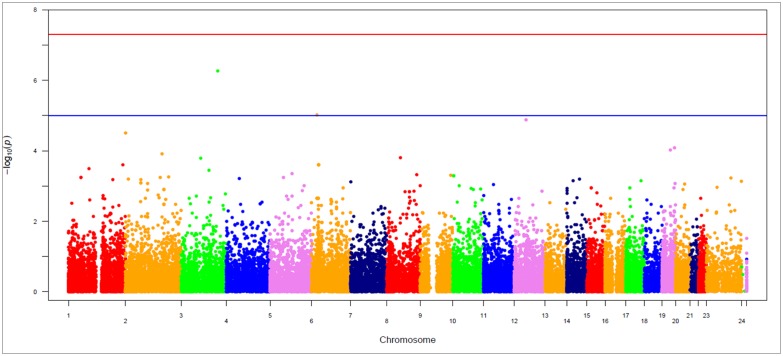
Manhattan plot of association for pathologic Gleason score upgrading in low risk prostate cancer from an analysis of 242,221 single nucleotide polymorphisms on a custom HumanExome BeadChip v1.0 (Illuminam Inc.). The blue line represents p = 1×10^−5^.

The genotype frequencies in both PGU and no PGU were analyzed using a logistic regression model (Fig. 1). Results from genotyping 242,186 SNPs on a custom HumanExome BeadChip 12v1-1 (Illumina Inc.) in blood DNA, showed that 15 SNPs (rs3795832, rs606149, rs4927635, rs3770657, rs61740794, rs3770655, rs12469465, rs33999879, rs1823068, rs117692893, rs3857984, rs12895416, rs4805162, rs641738 and rs1801164) were significantly associated with PGU in men with low-risk PCa (Table 2). The top five associations found for PGU were non-synonymous SNPs: rs33999879 (SMC4, Asn356Ser, OR = 0.07, P = 5.4×10^−7^), rs117692893 (KIAA0319, Ser255Thr, OR = 0.16, P = 9.7×10^−6^), rs641738 (TMC4, Gly17Glu, OR = 0.39, P = 8.1×10^−5^), and rs4805162 (ZNF565, Thr188Ile, OR = 0.43, P = 9.6×10^−5^) were negatively correlated with PGU. The rs4927635 (SNTG2, Thr495Met, OR = 2.69, P = 3.1×10^−5^) was positively correlated with PGU after logistic analysis. Another significant SNPs - rs61740794 (OR: 2.14), rs12469465 (OR: 2.16), and rs12895416 (OR: 2.21) were positively correlated with PGU, however all of the others were negatively correlated with PGU. Among these top SNPs, rs33999879 retained significance after the less strict correction for multiple testing, estimating a FDR of <50% for P<0.01, (See adjusted p-value in Table 2).

**Table 2:** Logistic regression analysis of exome genotyping with pathologic Gleason score upgrading in low risk prostate cancer patients.

SNPID	Chr	Gene	Alleles	Minor Allele Frequency		OR (95%CI)	p-value	Adjusted p-value
				No upgrade	Pathologic upgrade			
rs3795832	1	LRRC8B	G>A	0.157	0.054	0.31 (0.16–0.60)	0.0003178	0.6648
rs606149	1	-	A>G	0.435	0.266	0.47 (0.30–0.73)	0.0006623	0.6648
rs4927635	2	SNTG2	C>T	0.250	0.473	2.69 (1.67–4.34)	3.14E-05	0.2552
rs3770657	2	ETAA1	A>G	0.613	0.431	0.48 (0.31–0.74)	0.0008172	0.6867
rs61740794	2	ETAA1	G>A	0.343	0.527	2.14 (1.37–3.33)	0.0006518	0.6648
rs3770655	2	ETAA1	T>C	0.613	0.431	0.48 (0.31–0.74)	0.0008172	0.6867
rs12469465	2	-	C>T	0.343	0.530	2.16 (1.39–3.36)	0.0005517	0.6648
rs33999879	3	SMC4	A>G	0.093	0.007	0.07 (0.02–0.27)	5.38E-07	0.0175
rs1823068	5	PDE4D	A>G	0.349	0.192	0.44 (0.28–0.71)	0.0005653	0.6648
rs117692893	6	KIAA0319	A>T	0.113	0.020	0.16 (0.06–0.40)	9.66E-06	0.1433
rs3857984	9	-	C>T	0.443	0.269	0.46 (0.30–0.72)	0.0004935	0.6648
rs12895416	14	-	T>C	0.287	0.470	2.21 (1.39–3.50)	0.0006266	0.6648
rs4805162	19	ZNF565	G>A	0.537	0.333	0.43 (0.28–0.66)	9.58E-05	0.5199
rs641738	19	TMC4	C>T	0.343	0.170	0.39 (0.24–0.63)	8.11E-05	0.5199
rs1801164	23	IRS4	C>G	0.539	0.286	0.34 (0.18–0.64)	0.0005825	0.6648

Among 242,221 single nucleotide polymorphisms, only 15 SNPs significant to pathologic Gleason score upgrade were shown.

Adjusted p-value was calculated by multiple testing method of false discovery rate.

Abbreviations: OR: odd ratio; CI: confidence interval.

**Figure 2:**
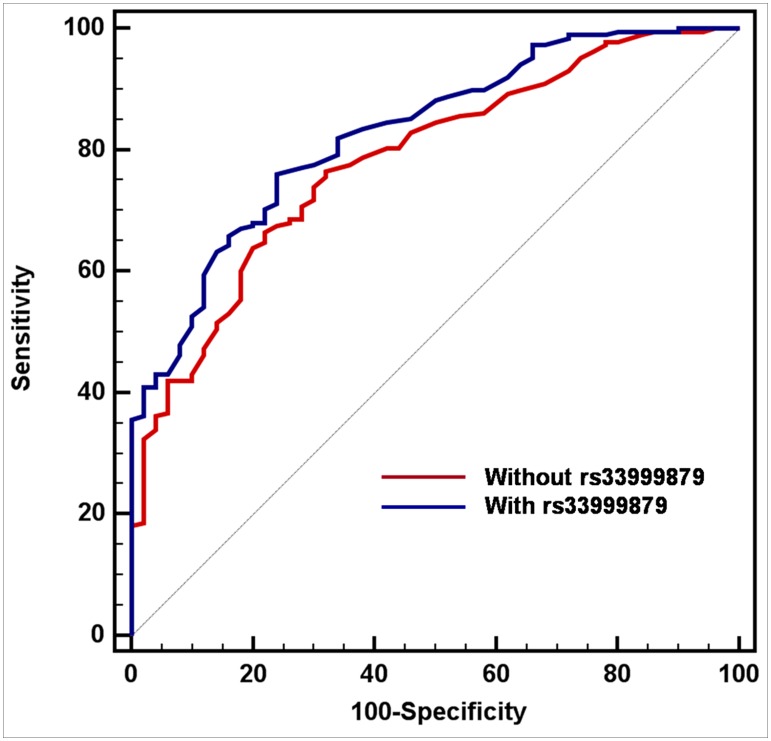
Receiver operating characteristics curves of the multivariate logistic regression model, which was devised for the pathologic Gleason score upgrading after radical prostatectomy with and without rs33999879 among low risk prostate cancer.

The Multivariate models incorporating the variables of age, PSA density, clinical stage, number of positive cores, and tumor percentage in cores which included and excluded rs33999879, are shown in Table 3. PSA density, number of positive cores, and tumor percentage in cores, were significant predictors of PGU in low-risk PCa patients who underwent RP. The predictive accuracies for the multivariate model, which included and excluded rs33999879, were 82.9% and 78.3%, respectively, among the low-risk PCa patients. Including rs33999879 in the model which consist of Epstein's criteria, significantly increased the predictive accuracy (95% CI: 0.0000737–0.0893, p = 0.0196) (Fig. 2).

**Table 3:** Multivariate logistic regression models of potential predictors for pathologic upgrading among the low risk prostate cancer patients.

	Multivariate logistic regression model not including rs33999879			Multivariate logistic regression model including rs33999879		
Variables	HR	95% CI	P value	HR	95% CI	P value
Age (yrs)	1.046	0.998–1.097	0.061	1.048	0.994–1.104	0.083
PSA density (ng/ml)	3.525	1.695–7.333	0.001	3.397	1.568–7.360	0.002
Number of positive core (≤2 vs 2<)	5.377	1.756–9.462	0.003	4.186	2.099–6.196	0.003
Tumor percent in cores (%)	1.027	0.998–1.057	0.071	1.028	0.997–1.059	0.059
Clinical stage (T1 vs T2a)	1.201	0.515–2.802	0.672	0.961	0.403–2.287	0.928
rs33999879	-	-	-	0.038	0.006–0.231	<0.001
Areas under curve of each models	**0.783**			**0.829**		

Abbreviations: HR: hazard ratio; CI: confidence interval; PSA: prostate specific antigen.

## Discussions

The present study was conducted to investigate the potential genetic biomarkers for PGU in low-risk PCa patients. Logistic regression analysis suggested that one SNP, (rs33999879), was significantly inversely associated with a risk for PGU in prostate cancer, when compared with those who did not experience PGU. Additionally, we applied this information from genetic studies to a real clinical model based on previously established factors, and we found an additional predictive gain in discovering CIPC.

AS is a promising option for PCa, to reduce active treatment related complications and to maintain quality of life, however there has been concern about delaying treatment [10], [11]. Therefore, the accurate selection of candidates for AS is very important, and numerous criteria have been introduced and validated [18]. Among them, the Epstein criterion for predicting CIPC is probably the most useful in the actual clinical setting. Currently, the Epstein criterion, which is used by NCCN, might arguably be the best tool for prediction of CIPC. However previously reported validation studies of the Epstein criteria showed somewhat disappointing results. Bastian et al. [19] showed that the Epstein criteria were inaccurate in predicting insignificant tumor in 16% of cases. Jeldes et al. [20] also showed that 24% of men included in the Epstein criteria had experienced pathological GS upgrading after RP in European cohorts. In Koreans, the same ethnicity as in this study, Lee et al. [21] showed 30.5% pathological GS upgrading after RP, in men who fulfilled the criteria of Epstein. More recently, Sundi et al. [11] showed 27.3% of African-American men with very low risk of PCa exhibited pathological GS upgrading.

To overcome this discordance between clinical criteria and real clinical outcomes, investigations into novel biomarkers to improve the ability to categorize PCa are essential [18]. Due to advances in understanding of the molecular biology of prostate carcinogenesis, multiple susceptibility genes, and many additional mechanisms involved in carcinogenesis and cancer progression have been discovered [22]. However, no single biomarker is able to improve on the common clinical parameters included in the currently used prediction models. The study by Haese et al. [23], investigated the use of PCA3 (prostate cancer antigen 3) testing in a rebiopsy setting of patients with a negative prostate biopsy. In their work, the ability to detect PCa risk increased with increasing PCA3 scores, and therefore PCA3 testing might be applicable to surveillance without active treatment. However, Deras et al. [24] showed that PCA3 was independent of tumor volume, which is why the true value of PCA3 currently remains unclear.

Recent genome-wide association studies (GWAS) of PCa have identified many regions in the genome harboring susceptibility alleles that confer risk for PCa. Eeles et al. [12] identified 23 new PCa susceptibility loci in a well-organized, large cohort, study. Schumacher et al. [25] showed that 2q37.3 (rs2292884) was a new susceptibility locus associated with overall PCa. However, these studies originated from case-control studies in which the control group was from a normal healthy population. Our study is the first aimed at identifying genetic markers to improve the predictive accuracy of PGU among homogenous men who underwent RP.

The rs33999879 SNP is located at 3q26.1 within the structural maintenance region of chromosome 4 (SMC4), which is critical for mitotic chromosome condensation and DNA repair. A previous study on SMC4 in liver cancer and lymphoma showed that SMC4 was associated with tumor size and the advanced stages of cancer [26], however there are no studies examining SMC4 and PCa. By bio-molecular investigation about SMC4 in prostate cancer, we should confirm the mechanism of SMC4 in PCa. The strengths of our study were that we applied information from genetics to a real clinical setting, adjusting previously established factors—such as PSA density, clinical stage, and biopsy tumor volume, which are factors in Epstein's criteria. After including the genetic information, the predictive power regarding PGU was significantly increased, therefore this genetic information may be an appropriate genetic marker to select patients for AS.

The present study had several limitations. Small sample size and discrepancy of each group represents one of them, however of all the men included in this study were from a homogenous racial population. The PCa diagnosed in Asian, American, and European men may have innate differences associated with racial and/or environmental factors. As PCa is hormone-dependent, various investigators have suggested that racial variations in the serum levels of testosterone, together with its derivatives, may contribute to differences in PCa risks and prognoses, among different races [27], [28]. Some have suggested that such differences in the hormonal milieu, in addition to a lack of PSA screening, may also play a role in the generally more aggressive profile of PCa diagnosed in contemporary Korean men, compared to their Western counterparts [29]. These effects of racial difference may have their origins in genetic differences; therefore, our data from an Asian population may differ from that from study of Western population. Another limitation was high number of PGU in low-risk PCa patients. The single pathologist who has a specialty for uro-oncology reviewed all of the specimens included in this study through International Society of Urological Pathology (ISUP) recommendation of modified Gleason score which announced in 2005 after handling by very thin sectioned. Regardless the extent of tumor, any Gleason pattern 4 was found in any section at radical prostatectomy specimen with 99% Gleason pattern 3, therefore Gleason score was 3+4. In our results, 174 patients (85.7%) were pathologically upgraded to Gleason score 3+4 and only 29 patients (14.3%) to Gleason score 4+3 among 203 patients had experienced PGU after RP. Previous our hospital data showed 30.5% PGU among clinical insignificant prostate cancer [21], however newly reviewed Gleason scoring system had trend a higher rate of PGU after RP. Despite these limitations, the potential predictive marker should be judged on its capacity to improve the pre-existing optimized predictive model rather than simply on its status as an independent variable [30]. Our finding is useful for patients and clinicians who deal with complex treatment decisions, and we may have identified a novel, clinically useful, biomarker, which will of course have to be validated in a large scale, multiracial study.

## Conclusions

We showed that rs33999879 was a significant predictor of PGU, and that the addition of genetic information from the exome sequencing effectively enhanced the predictive accuracy of the multivariate model, which incorporated various factors including criteria for AS. These results should be validated in a future study, and this could lead to an accurate model that enables suitable CIPC patients to be selected for AS.
